# Depressive Symptoms among Patients with Diabetes in Qatar: Frequency and Potential Determinants

**DOI:** 10.3390/healthcare9030302

**Published:** 2021-03-09

**Authors:** Hiba Bawadi, Alanood Al-Shahwani, Dana Arafeh, Daniah Al-Asmar, Joyce Moawad, Zumin Shi, Suhad Daher-Nashif

**Affiliations:** 1 Department of Human Nutrition, College of Health Sciences, QU-Health, Qatar University, Doha 2713, Qatar; hbawadi@qu.edu.qa (H.B.); aa1609206@student.qu.edu.qa (A.A.-S.); da1609127@student.qu.edu.qa (D.A.); da1703795@student.qu.edu.qa (D.A.-A.); jmoawad@qu.edu.qa (J.M.); zumin@qu.edu.qa (Z.S.); 2 Population Medicine Department, College of Medicine, QU-Health, Qatar University, Doha 2713, Qatar

**Keywords:** diabetes, depression, Qatar Biobank, risk behaviors, determinants of health

## Abstract

*Background:* Diabetes is a highly prevalent chronic disease that is associated with major complications. Findings regarding risk of depression among patients with diabetes are controversial. This study aimed to determine the prevalence and determinants of depressive symptoms among Qatari patients with type 2 diabetes. *Methods:* This cross-sectional study was based on Qatar Biobank (QBB) data of 2448 Qatari adults with diabetes aged 21–60 years old. Data regarding age, gender, education, income, body mass index (BMI), medication use, glycated hemoglobin (HbA1c) were retrieved. Patients’ responses to the Patient Health Questionnaire-9 (PHQ-9) were also obtained. Data analyses was performed using STATA 16, and statistical significance was considered at a *p*-value of <0.05. *Results:* Of the 2448 participants, 15.4% (*n* = 378) had self-reported depressive symptoms. Depressive symptoms were frequent among females (69.6%), smokers (15.9%), and participants with a higher level of education (47.1%). Average age of participants who reported depressive symptoms was significantly less that among participants without depressive symptoms (44.8 vs. 52.9 years). Qatari women with diabetes seem to be at higher risk of depression when compared to men (OR = 1.819, 95% CI: 1.42–2.33); The odds of reporting depressive symptoms were 35% higher among patients with more advanced educational qualifications (OR = 1.351, 95% CI: 1.00, 1.82). Smokers were twice as likely to report depressive symptoms as their non-smoking counterparts. There was no significant relationship between depression and poor glycemic control, physical activity, BMI, or insulin use. *Conclusions:* In summary, the study results suggest that several sociodemographic factors, such as age, gender, and level of education were associated with the risk of depressive symptoms among Qataris with diabetes.

## 1. Introduction

Diabetes is defined as a heterogeneous metabolic disorder that is characterized by high blood glucose resulting from either impaired insulin sensitivity, secretion, or both [1]. It is associated with various micro- and macro-vascular complications if left untreated, in addition to social and financial burden [2,3]. Globally, diabetes has become one of the most dramatic problems among all current human health issues [4]. In 2019, it was estimated that approximately 463 million people are diagnosed by diabetes, which is expected to increase to 578 million people in 2030 and then to 700 million by 2045 [5]. The prevalence of diabetes in Qatar was 17% in 2019, and it is predicted that it will account for 7% of the total disease burden [6].

Many epidemiological studies have shown a strong relationship between chronic diseases occurrence and treatment with the mental health of the patients [7]. In fact, depression has been found to be associated with the increased complications of several chronic diseases including diabetes, heart disease, hypertension, cancer, and obesity [8]. It was reported that several unhealthy behaviors were commonly associated with occurrence of depression and poor diabetes management including physical inactivity, smoking, and consumption of high caloric diets [9,10].

Findings regarding the association between depression and diabetes control are controversial. Several research studies were conducted to investigate the bi-directional relationship between depression and diabetes [11]. Most of these studies indicate that depression is a stronger causative factor of diabetes than the opposite [12]; whereas depression increases the risk of type 2 diabetes by 60%, the latter is only associated with a modestly increased risk of depression [13]. On the other hand, others found that patients with diabetes have increased risk of developing depression. A meta-analysis showed that mental illness is associated with hyperglycemia in patients with diabetes (type 1 and type 2) [14], In addition, a systematic review and meta-analysis study underlined that patients with type 2 diabetes exhibited a slightly increased risk of developing depression [13]. Conversely, other research uncovered a negative association between depression and type 1 diabetes that highlighted low rates of depression and major depressive disorders in adults with type 1 diabetes [15]. The present study aimed to determine the frequency and determinants of depressive symptoms among patients with type 2 diabetes in Qatari.

## 2. Methods

### 2.1. Study Design and Population

A cross-sectional from secondary data collected by Qatar BioBank (QBB) [16]. The QBB data collection started 2012 and reached a total number of participants of 15,000 in 2018. Data of 2448 patients with type 2 diabetes were retrieved from QBB. Sociodemographic data and depressive symptoms were collected by self-administrative questionnaires. Health data were collected by a nurse interview and nurse health examination. All data collection and biological samples collection were ethically approved from Hamad Medical Corporation Ethics Committee (Ex-2021-QF-QBB-RES-ACC-00003-0152). The use of data for the current study was approved from QBB. 

### 2.2. Outcome Variable: Depressive Symptoms

Data regarding the occurrence of depressive symptoms were collected by PHQ-9 questionnaire. The questionnaire is a self-report measure that is mainly used for screening not diagnostics purposes. It includes nine statements of symptoms with four possible responses of frequencies. Each statement is scored from zero (not at all) to three (nearly every day). The total calculated score is based on the summation of 9 scores. The final total PHQ-9 score indicates the severity of depressive symptoms. A score of 0–4 indicates no or minimal depression, 5–9 points equates to mild depression, 10–14 points highlights there being moderate depression, and 15–19 points is indicative of severe depression [17]. A PHQ-9 score of ≥10 was used to define an incidence of the depressive symptoms.

### 2.3. Exposure Variables

Age, gender, income and educational level (low = secondary school or less; medium = high school; high = professional qualifications and above) were collected by a self-administered questionnaire. Smoking status of contributors was also self-reported in the questionnaire. Participants were asked to choose from the following answers: 1 = nonsmoker, 2 or 3 = smoker on most days or occasionally, 4 or 5 = ex-smoker, having stopped smoking or having tried once or twice to do so. Missing answers led to the assumption that a non-smoker was answering the questionnaire as the majority of contributors were women. Physical activity was assessed by the international physical activity questionnaire (IPAQ). 

Participants’ body weight and height were measured by a registered nurse. Participants were asked to remove their shoes and to stand on an electronic weighing scale. Height was measured by asking them to stand straight without shoes on against a wall. Body mass index was calculated. A BMI of 25–29.9 kg/m^2^ was defined as overweight, and a BMI of ≥30 kg/m^2^ led to an individual being classified as obese. BMI was calculated via body weight being divided by meters of height squared (kg/m^2^).

The glycemic control of patients was assessed by HbA1c levels. Data regarding the use of medications, such as insulin, diabetes medication other than insulin, and hypertension medication, were collected by a registered nurse through face-to-face interviews.

### 2.4. Statistical Analysis

The chi-square (χ2) test was used for categorical variables, and ANOVA was employed with continuous variables. To assess the association between depression among people with diabetes and deferent sociodemographic determinants, multivariable logistic regression was utilized. In relation to this, three different models were used: model one was adjusted for age, gender, and poor glycemic control; model two was further attuned for smoking, physical activity, education, and BMI; lastly, model three was further adjusted for insulin use, utilization of diabetes medications other than insulin, and the use of hypertension medications. Data analyses were performed using STATA 16 (Stata Corporation, College Station, TX, USA), and statistical significance was considered at a *p*-value of <0.05.

## 3. Results

The study showed that out of 2448 participants, 378 (15.4%) reported depressive symptoms. Patients with self-reported depressive symptoms had lower average age than those with no symptoms (44.8 vs. 52.9 years, respectively). There was a statistical gender differences with regards to the prevalence of depressive symptoms. The number of Qatari women with the depressive symptoms (69.6%) was higher than that in men (30.4%). Higher percentage of patients with depressive symptoms received high education (*p* < 0.001). The average BMI of patients with self-reported depressive symptoms was higher than that of patients with no reported symptoms (32.7 vs. 32.0 kg/m^2^, respectively). Patients with no reported depressive symptoms had poor glycemic control (52.3%) in comparison with those who self-reported depressive symptoms (44.2%). The average HbA1c value in the study sample was 7.4 ± 1.8% (Table 1).

Table 2 shows the odds ratio (95% CI) for depressive symptoms among Qatari adults with diabetes resulted from different adjusted logistic regression models. Model 1 shows association between age (OR = 0.943; 95% CI: 0.93–0.95), gender -being a women (OR = 1.81; 95% CI: 1.42–2.33) and risk of depressive symptoms. The association between risk of depressive symptoms and younger age and female gender were attenuated as determinants for the presence of depressive symptoms after controlling for smoking, physical activity, education, BMI status, insulin use, diabetes medication other than insulin, and hypertension medication use.

Our data showed that there was a significant relationship between smoking and the presence of depressive symptoms. The adjusted odds of reporting depressive symptoms was doubled among smokers as compared to their non-smokers (OR = 1.81; 95% CI: 1.42–2.33). Meanwhile, ex-smokers had 56% higher risk of depressive symptoms as compared to non-smoking, however this association was not attenuated after controlling for diabetes and hypertension medication use. The odds of reporting depressive symptoms among patients who do not take diabetes medication were 73% higher than those who take diabetes medication. In the fully adjusted model, no associations were found between glycemic control, physical activity, education, body mass index and insulin use. 

## 4. Discussion

This study aimed to uncover the prevalence of depression amongst Qatari patients with type 2 diabetes, and to investigate the possible sociodemographic determinants of depression in the target population. Around 15.4% of the study sample reported suffering from depressive symptoms. This finding was in line with findings on the general population of Qatar [18]. The current study revealed several sociodemographic and health determinants of reported depressive symptoms among patients with diabetes, including younger age; female gender, smoking, obesity, having higher education degree, and the use of diabetes and hypertension medications. 

This study found that younger patients were more likely to report depressive symptoms. Similar results were reported previously in the literature [19,20,21]. Andreoulakis et al. (2012) reported that younger age was independently associated with persistence of major depression over time, and was independently associated with persistence of elevated depressive symptoms [20]. Furthermore, an observational study using the German diabetes database (Diabetes-Patienten-Verlaufsdokumentation—DPV) and searched for patients up to the age of 25 years in Germany, found correlations between being young and showing depressive symptoms among patients with diabetes [21]. This finding can be explained by the perception of diabetes impact on the quality of life of younger adults as compared to that on older adults which may lead to deterioration of their mental health [22]. 

Female gender among patients with diabetes was also a factor associated with increased risk for depression. Several studies reported similar finding [20,21,23,24]. A recent study found that women with Type 2 diabetes, are twice as likely to be diagnosed with depression compared with men [25]. Another study found a higher incidence of depression among women with diabetes was higher than that among men with diabetes [21]. Similar findings were reported in a cross sectional study conducted in Pakistan [26]. general Qatari population, females were also at higher risk of depression as compared to men [18]. It was found that Qatari women were at a higher risk of developing depression (53.% vs. 46.9%) and anxiety disorder (56.7% vs. 43.3%) as opposed to men [27]. Deischinger et al., explain that adult women are about two times more likely to be diagnosed with depression, have a higher prevalence of MDD due to biological factors and the psychological burden of the disease and/or men being underdiagnosed with depression [25].

Smoking was associated with depressive symptoms. This association was reported in past studies [21,28]. Solberg et al., found that smokers with diabetes were more likely to report fair or poor health and that they often feel depressed. The association may be explained by the lack of active engagement of smokers in self-care or to adherence to diabetes care recommendations [28].

This study did not find association between obesity and risk of depressive symptoms among patients with diabetes. However, previous studies reported such association. Previous studies on the general Qatari population found obesity and hypertension to be the central risk factors for diabetes [29,30]. Indeed, depression was recognized to be strongly associated with obesity among Middle Eastern societies, including Qatar [31]. Indeed, one specific study demonstrated that obesity was one of the major predictors for depression and anxiety among patients with diabetes in Qatar [30]. Two divergent schools of thought can explain these findings. The first one highlights behavior and suggests that emotional stress, such as that experienced when depressed and/or anxious, results in people adopting unhealthy lifestyle habits, which in turn leads to diabetes. The second option is physiological, which is where emotional stress causes biochemical changes to occur in the body, meaning that there is a growth in abdominal obesity that subsequently leads to diabetes [32].

The association between level of education and reported depressive symptoms was not consistent across logistic regression models with different levels of adjustments. We found that receiving a college education was linked to higher percentages of reported depressive symptoms. Similar finding was reported by past studies [21]. Collins et al., found that educational attainment was associated with a higher depression score, while this was not the case in anxiety scores [33]. Based on the HADS, Collins et al., reported and evidence of high levels of anxiety and depression symptoms in patients with diabetes; 22.4% (95% confidence interval = 20.2–24.7%) exceeded the HADS cut-off score of ‘mild to severe’ depression. [33].

Other researchers found the opposite association, and reported higher risk of depression among populations with lower educational levels [10,20,24].

A study from Malesia found depression in the diabetes population has been associated with potential sociodemographic and clinical factors. Age, socioeconomic status, and education level, were important correlates for major depression among people with diabetes [34].

In addition to sociodemographic factors and health risk behaviors, use of medication was associated with the risk of depressive symptoms. The majority of Qatari patients with diabetes who take diabetes medications other than insulin have lower odds of depression as opposed to those who do not. A previous study reported that adherence to diabetes medication may be considered as a preventive factor to depression [35]. It was also reported that depressive mood is a barrier to diabetes management, i.e., the relationship between adherence to diabetes medications and depression works in both directions. We did not find an association between insulin use and depression. Nevertheless, some work in this area has found a link between taking insulin and depression among patients with diabetes. An examination of impact of using insulin on developing major depressive disorder among people with type 2 diabetes, found a higher prevalence of depression among patients using insulin [10]. Another study found a higher severity of depression among patients with diabetes who use insulin [17]. These gaps in the results may be due to differences in doses and types of combinations with other diabetes medications. 

The use of hypertension medications was also associated with risk of depressive symptoms. We found that 35.8% of participants who take hypertension medications have lower odds of depression in comparison to those who do not. This finding is in line with past studies. Among other investigations, a cross-sectional study showed that having hypertension was one of the factors strongly associated with anxiety and depression among patients with diabetes [36]. Authors also reported that using hypertension medication reduces the probability of depression among individuals with diabetes. This link explained by Hu et al., who found descriptions of increased prevalence of hypertension in depressed patients, increased prevalence of depression in hypertensive patients, association between depressive symptomatology and hypotension, and alteration of the circadian variation of blood pressure in depressed patients. There is considerable evidence suggesting that hyperreactivity of the sympathetic nervous system and genetic influences are the underlying mechanisms in the relationship between depression and hypertension [37].

Our study had several limitations, such as its cross-sectional design; therefore, we cannot come to any solid conclusions about the causation of this association between depression and diabetes. Another pitfall in this research was that the former condition was self-reported using the PHQ-9, which is a screening tool not a diagnostic tool for diabetes. Conversely, this study’s main strength was its use of detailed data about sociodemographic factors, health risk behaviors, and medication use. In conclusion, the current study identified several risk factors for depression in patients with diabetes in Qatar including age, gender, and education, smoking and medication use.

In conclusion, diabetes and depressive symptoms co-exist. Several determinants of depressive symptoms among Qatari patients with diabetes were revealed including younger age, female gender, and use of diabetes and hypertension medication. Future studies to further understand directions of these relationships so that appropriate interventions are introduced to prevent progression of these symptoms to clinical depression.

## Figures and Tables

**Table 1:** Sample characteristics by presence pf depressive symptoms.

	Total	No	Yes	*p*-Value
	*N* = 2448	*N* = 2070	*N* = 378	
Age (years)	51.6 ± 11.9	52.9 ± 11.5	44.8 ± 11.8	<0.001
Gender				<0.001
Men	1000 (40.8%)	885 (42.8%)	115 (30.4%)	
Women	1448 (59.2%)	1185 (57.2%)	263 (69.6%)	
Education				<0.001
Low	982 (40.1%)	882 (42.6%)	100 (26.5%)	
Medium	461 (18.8%)	361 (17.4%)	100 (26.5%)	
High	1004 (41.0%)	826 (39.9%)	178 (47.1%)	
Smoking				0.035
Non-smoker	1820 (74.3%)	1546 (74.7%)	274 (72.5%)	
	296 (12.1%)	236 (11.4%)	60 (15.9%)	
Ex-smoker	332 (13.6%)	288 (13.9%)	44 (11.6%)	
HbA1C %	7.4 ± 1.8	7.5 ± 1.8	7.2 ± 2.0	0.019
Poor glycemic control	1250 (51.1%)	1083 (52.3%)	167 (44.2%)	0.004
Leisure time physical activity (MET hours/week)	12.6 ± 36.4	12.5 ± 37.3	13.2 ± 31.1	0.74
BMI (kg/m^2^)	32.1 ± 6.0	32.0 ± 5.8	32.7 ± 6.8	0.036
BMI categories				0.71
Normal	212 (8.7%)	178 (8.6%)	34 (9.0%)	
Overweight	751 (30.7%)	642 (31.1%)	109 (28.9%)	
Obese	1481 (60.6%)	1247 (60.3%)	234 (62.1%)	
Insulin use	621 (25.4%)	529 (25.6%)	92 (24.3%)	0.62
Diabetes medication other than insulin	1705 (69.6%)	1511 (73.0%)	194 (51.3%)	<0.001
Hypertension medication use	844 (34.5%)	742 (35.8%)	102 (27.0%)	<0.001

Continuous variables presented as mean ± standard deviations. Categorical variables were represented as *n* (%).

**Table 2:** logistics regression for factors associated with the presence of depressive symptoms Odds ratio [95% CI].

	Model 1	Model 2	Model 3
Poor glycemic control	0.920 [0.73, 1.16]	0.918 [0.72, 1.16]	1.029 [0.79, 1.35]
Age	0.943 *** [0.93, 0.95]	0.946 *** [0.94, 0.96]	0.948 *** [0.94, 0.96]
Women	1.819 *** [1.42, 2.33]	2.557 *** [1.80, 3.63]	2.458 *** [1.73, 3.49]
Smoking			
Non-smoker		Ref	Ref
Smoker		2.098 *** [1.38, 3.19]	2.030 *** [1.33, 3.09]
Ex-smoker		1.561 * [1.01, 2.40]	1.502 [0.98, 2.31]
Physical activity			
Tertile 1		Ref	Ref
Tertile 2		0.895 [0.63, 1.27]	0.915 [0.64, 1.30]
Tertile 3		0.824 [0.63, 1.08]	0.811 [0.62, 1.06]
Education			
Low		Ref	Ref
Medium		1.298 [0.91, 1.85]	1.303 [0.91, 1.86]
High		1.351 * [1.00, 1.82]	1.323 [0.98, 1.79]
BMI status			
Normal		Ref	Ref
Overweight		1.030 [0.66, 1.62]	1.048 [0.66, 1.65]
Obese		1.166 [0.76, 1.79]	1.192 [0.77, 1.85]
Insulin use			0.876 [0.64, 1.19]
Diabetes medication other than insulin			0.575 *** [0.44, 0.75]
Hypertension medication use			1.370 * [1.03, 1.83]

Exponentiated coefficients; 95% confidence intervals in brackets. * *p* < 0.05, *** *p* < 0.001.
